# Two new *Meiogyne* species from Malesia and new combinations in *Meiogyne* and *Monoon* (Annonaceae) in the Asia-Pacific region

**DOI:** 10.3897/phytokeys.272.175385

**Published:** 2026-03-20

**Authors:** Ming-Fai Liu, Jérôme Munzinger, Junhao Chen

**Affiliations:** 1 Flora Conservation Department, Kadoorie Farm & Botanical Garden, Lam Tsuen, Lam Kam Road, New Territories, Hong Kong SAR, China Department of Biological Sciences, National University of Singapore Singapore Singapore; 2 AMAP, Université Montpellier, IRD, CIRAD, CNRS, INRAE, Montpellier, France AMAP, Université Montpellier, IRD, CIRAD, CNRS, INRAE Montpellier France; 3 Botanical Research, Singapore Botanic Gardens, 1 Cluny Road, Singapore 259569, Singapore Flora Conservation Department, Kadoorie Farm & Botanical Garden Hong Kong China; 4 Department of Biological Sciences, National University of Singapore, 16 Science Drive 4, Singapore 117558, Singapore Singapore Botanic Gardens Singapore Singapore

**Keywords:** Annonaceae, Borneo, *Meiogyne cylindrocarpa*, New Guinea, Vanuatu

## Abstract

Two new species of *Meiogyne* (Annonaceae) are described from Malesia based on morphological evidence and the results of a recent molecular phylogenetic study: *Meiogyne saundersii* from Peninsular Malaysia, Sumatra, and Borneo, and *Meiogyne stenophylla* from New Guinea. *Meiogyne saundersii* is morphologically similar to *M. cylindrocarpa* but differs in its leaf apex (abruptly caudate vs. acute, rarely shortly acuminate) and inner petal corrugation (tentacular vs. weakly longitudinally grooved). *Meiogyne stenophylla* is unique in the genus in its extremely narrow leaves (< 2 cm wide) with an attenuate apex; it is also similar to *M. cylindrocarpa* but differs in its lanceolate (vs. broadly ovate) petals and weakly warty (vs. weakly longitudinally grooved) outgrowth on the adaxial surface of the inner petals. In addition, two new combinations are proposed, *Meiogyne wilsonii* and *Monoon papuanum*, based on the results of the most recent molecular phylogeny of *Meiogyne* and related genera.

## Introduction

*Meiogyne* Miq. (Annonaceae, Malmeoideae, Miliuseae, Sapranthinae) is a genus of trees and treelets found in tropical and subtropical forests (van Heusden 1994; Thomas et al. 2012; Nge et al. 2024). Its distribution ranges from India to the Western Pacific, reaching as far east as Fiji and Tonga (Xue et al. 2014; Turner and Utteridge 2017). The diagnostic features of *Meiogyne* are the verrucose growth or corrugation on the adaxial surface of the inner petals and the elongated anther connectives of the innermost stamens (van Heusden 1992, 1994; Johnson et al. 2019). The inner petal corrugation appears to serve different functions depending on the pollination system of the species. In *Meiogyne* species that offer some form of reward to their pollinators, the inner petal corrugation has been demonstrated to function as a food body (Liu et al. 2025a) or brood site for pollinators (Liu et al. 2025b). *Meiogyne heteropetala* (F.Muell.) D.C.Thomas, Chaowasku & R.M.K.Saunders bears dark maroon flowers that imitate aerial litter, and it was postulated that the tactile cues of the corrugation may play a role in oviposition-site mimicry (Liu et al. 2025c). The inner petal corrugation has independently evolved multiple times in Annonaceae (van Heusden 1992; Xue et al. 2017a), likely contributing to the taxonomic confusion of *Meiogyne* with related genera such as *Pseuduvaria* (Wang et al. 2021).

Since the monographic revision of the genus by van Heusden (1994), the taxonomy of *Meiogyne* has undergone several changes. Based on morphological evidence, van Heusden (1994) amalgamated five now obsolete genera, *Ancana* F.Muell., *Ararocarpus* Scheff., *Guamia* Merr., *Polyaulax* Backer, and *Chieniodendron* Tsiang & P.T.Li, with *Meiogyne*. Turner and Utteridge (2015) noted the similarity of *Oncodostigma leptoneurum* Diels to *Meiogyne* and consequently transferred the species to *Meiogyne*. With the transfer of the remaining orphan species, *Oncodostigma microflorum* H.Okada, *Oncodostigma* Diels is entirely subsumed within *Meiogyne* (Xue et al. 2017b). In addition, molecular evidence within the last two decades has indicated that *Fitzalania* F.Muell. is nested within *Meiogyne* with strong support, unequivocally supporting the transfer of the genus *Fitzalania* to *Meiogyne* (Mols et al. 2004; Thomas et al. 2012; Xue et al. 2014). Although *Fitzalania* predates *Meiogyne*, the latter was successfully conserved against the former (Chaowasku et al. 2011; Xue et al. 2014).

Van Heusden (1994) identified three species with unusually broad distributions and/or wide variation in vegetative and reproductive characters, namely *Meiogyne cylindrocarpa* (Burck) Heusden, *Meiogyne stenopetala* (F.Muell.) Heusden, and *Meiogyne virgata* (Blume) Miq., and attributed their broad morphological variation to habitat adaptation. Molecular phylogenetic studies (Thomas et al. 2012; Xue et al. 2014; Jaikhamseub et al. 2022; Liu et al. 2025d) have since revealed that these taxa were indeed polyphyletic, resulting in the elevation of *Meiogyne cylindrocarpa* subsp. trichocarpa Jessup and *Meiogyne stenopetala* subsp. insularis (A.C.Sm.) Heusden to species rank as *Meiogyne trichocarpa* (Jessup) D.C.Thomas & R.M.K.Saunders and *Meiogyne insularis* (A.C.Sm.) D.C.Thomas, B.Xue & R.M.K.Saunders, respectively (Xue et al. 2014), and the resurrection of *Meiogyne subsessilis* (Ast) J.Sinclair in the *Meiogyne virgata* complex (Jaikhamseub et al. 2022).

**Figure 1.**
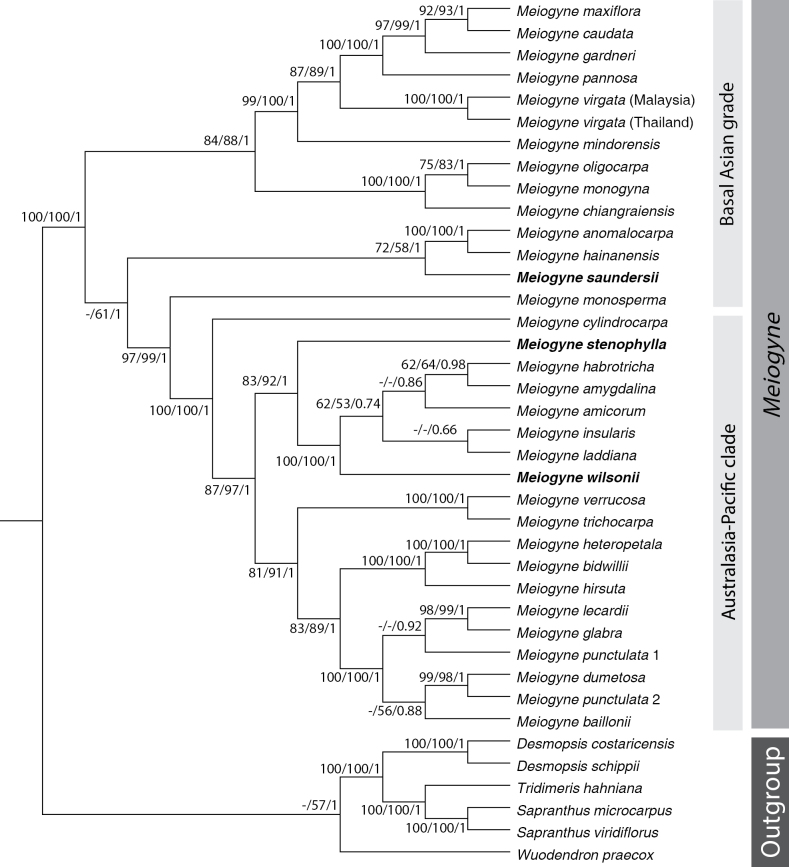
Bayesian 50% majority-rule consensus tree based on seven chloroplast DNA (cpDNA) regions (*matK*, *ndhF*, *ndhF–rpl32*, *rbcL*, *rpl32–trnL*, *trnL–F*, and *ycf1*) and 11 nuclear DNA (nDNA) regions (*ATPQ*, *COX6A*, *CHER1*, *DDB2*, *DJC65*, *EIF3K*, *MNJ7.16*, *NIA*, *PDF5*, *STG1*, and *MHF15.12*). Maximum parsimony bootstrap values (BS_MP_), maximum likelihood bootstrap values (BS_ML_), and Bayesian posterior probabilities (PP) are denoted at internal nodes in that order; “−” is annotated when BS_MP_, BS_ML_, or PP values < 50%. Adapted from Liu et al. (2025d).

Liu et al. (2025d) generated the most robust and well-resolved phylogeny of the genus to date based on seven chloroplast and 11 nuclear DNA regions. It was revealed that the genus comprises an Australasia-Pacific clade (APC) and a basal Asian grade (Fig. 1). The basal Asian grade contains three major lineages occurring in India, continental Southeast Asia, West Malesia, and the Philippines. The APC contains five major lineages, with three Australian clades and two Pacific clades. Furthermore, Liu et al. (2025d) revealed that *Meiogyne cylindrocarpa* remains polyphyletic and that two additional species should be segregated from it. The study also revealed the phylogenetic position of an undescribed *Meiogyne* species and the errant placement of a species within *Meiogyne*. The morphology of these taxa was examined in detail, and the formal taxonomic changes are provided here. In total, we propose two new combinations and describe two new species.

## Materials and methods

We examined herbarium specimens of *Meiogyne*, particularly those from Malesia and Melanesia, from the following herbaria: BRUN, K, KEP, L, P, SAN, SAR, and SING (acronyms follow Thiers, continuously updated), as well as high-resolution images of specimens (particularly types) from JSTOR Global Plants (https://plants.jstor.org/). For type specimens, an exclamation mark (!) indicates that either the physical specimen or a digital image was examined. The general plant descriptive terminology follows Beentje (2016). All measurements were taken from dried herbarium specimens, and the color of organs was derived from the collection label and observations of living material.

The conservation status of the species was evaluated using the criteria of the IUCN Red List (IUCN 2012; IUCN Standards and Petitions Committee 2024). The extent of occurrence (EOO) and area of occupancy (AOO) of each new species were calculated using the default 2 km^2^ grid in GeoCAT (Bachman et al. 2011; http://geocat.iucnredlist.org). The abbreviations used in the conservation assessments follow IUCN (2012).

## Taxonomic treatment

### Meiogyne saundersii

Taxon classificationPlantaeMagnolialesAnnonaceae

Junhao Chen & M.F.Liu sp. nov.

EB75D3D3-04AF-5B1A-B820-213EB26F61B7

urn:lsid:ipni.org:names:77377868-1

Figs 2A, 3

Meiogyne cylindrocarpa auct. non (Burck) Heusden: van Heusden (1994), *pro parte*; Turner (2014); Thomas et al. (2012) and Xue et al. (2014) as ‘Meiogyne cylindrocarpa subsp. cylindrocarpa 1’; Xue et al. (2021) and Jaikhamseub et al. (2022) as ‘Meiogyne cylindrocarpa 1’; Liu et al. (2025d) as ‘Meiogyne cylindrocarpa (Borneo)’.

#### Type.

Brunei • Temburong District: Kuala Belalong, Batu Apoi Forest Reserve, at the Kuala Belalong Fields Studies Centre, at the Sungai Belalong River upstream along path starting behind the houses, 4°33'N, 115°09'E, 7 Nov 1991, fl., *C. Hansen 1511* (holotype: BRUN [acc no. B004935!]; isotypes: K [K003311883!], L [L0047321!]).

#### Diagnosis.

Similar to *Meiogyne cylindrocarpa* in its relatively small leaves (typically < 10 cm long), indistinct leaf venation and cylindrical monocarps, but differs in its leaf apex (abruptly caudate vs. acute, rarely shortly acuminate) and inner petal corrugation (tentacular vs. weakly longitudinally grooved). See notes for additional minor differences in fruit morphology and habit.

**Figure 2.**
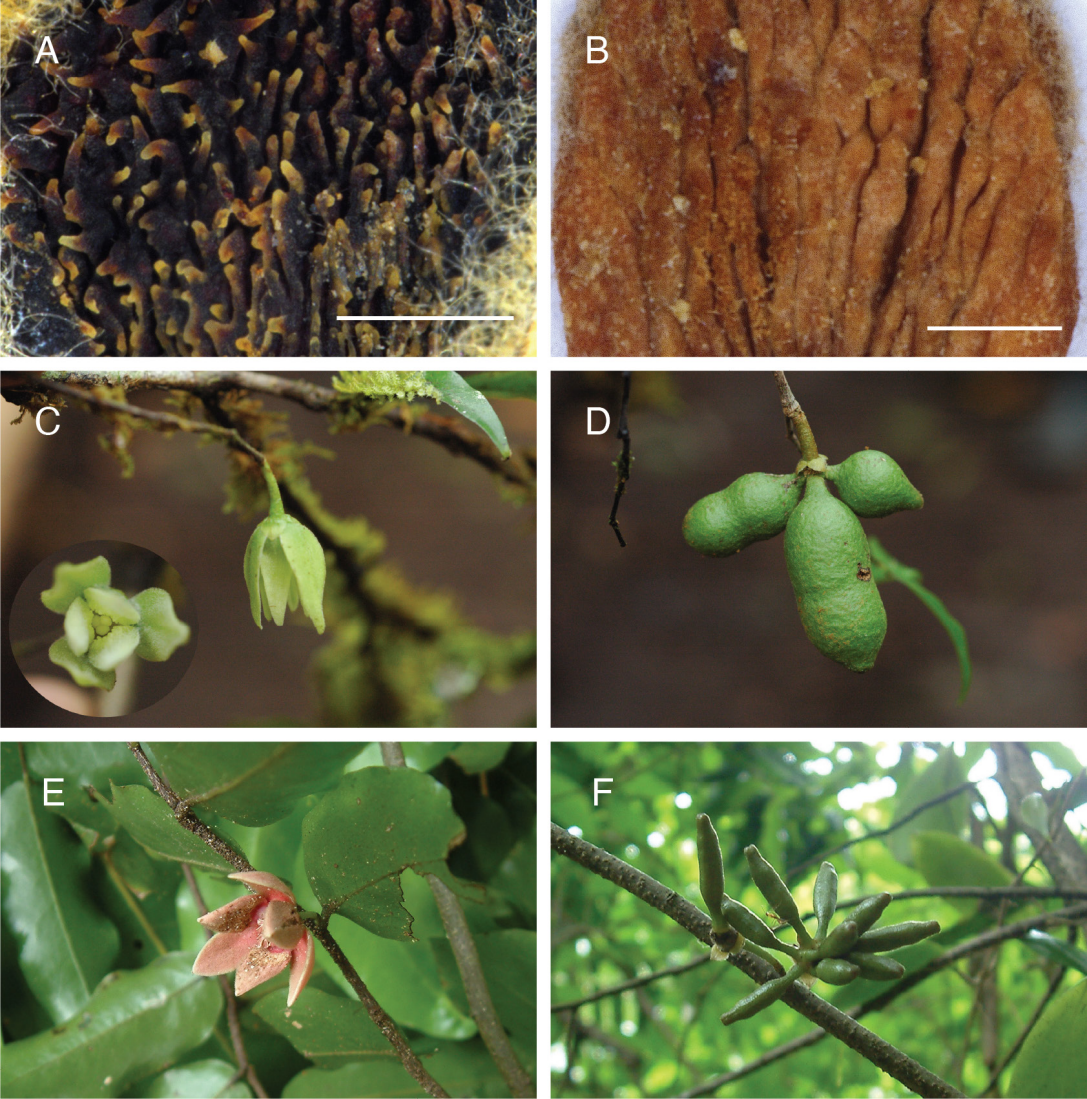
**A**. Adaxial inner petal outgrowth of *Meiogyne saundersii* Junhao Chen & M.F.Liu (*C. Hansen 1511*); **B**. Adaxial inner petal outgrowth of *Meiogyne cylindrocarpa* (Burck) Heusden (*Thomas 3418*); **C, D**. *Meiogyne stenophylla* M.F.Liu & Munzinger (*Munzinger et al. 6945*) flower and fruit, reproduced from Munzinger et al. (2020); **E, F**. *Meiogyne wilsonii* (Guillaumin) M.F.Liu & Munzinger (*Munzinger et al. 3589*) flower and early developing fruit. Scale bars: 1 mm. Photos: **A, B**. J. Chen; **C–F**. J. Munzinger.

#### Description.

Tree or treelet 5–20 m tall, 8–25(–60) cm dbh, stilt roots or buttresses absent; bark greyish green to dark brown, smooth, rarely rugose. Twigs with dense minute short hairs when young, glabrescent, sometimes with raised lenticels. Leaves chartaceous to subcoriaceous, blades elliptic, (3.8–)5.1–10.6 cm long, (1.5–)2–3.2(–3.6) cm wide, base cuneate, apex abruptly caudate, the acumen (0.5–)1–2.4 cm long, glabrous adaxially except minute erect hairs on the midrib when young, glabrous to sparsely appressed hairy abaxially; midrib sunken adaxially, raised abaxially, secondary and tertiary venation indistinct; petiole 2–4 mm long. ***Inflorescences*** axillary, 1-flowered; pedicel 4–7 mm long, 0.8–1 mm thick, densely appressed or short erect hairy, with 2–3 small bracts at the proximal end of the pedicel, sometimes with a medial bract. ***Buds*** conical, apex acute or obtuse. ***Sepals*** free, broadly ovate, 1–1.5 mm long, 1–2 mm wide, apex acute or obtuse, densely appressed hairy abaxially. ***Petals*** dull yellow except the dull purplish center, apex acute, sometimes rounded; outer petals triangular, 9–16 mm long, 3–4.5 mm wide, densely woolly hairy adaxially, sparsely appressed hairy abaxially; inner petals elliptic to ovate, 6–10.5 mm long, 4–5.5 mm wide, densely woolly hairy on both sides except the adaxial corrugated base, with a glabrous, tentacular corrugated patch on proximal 7/10–9/10 of adaxial surface. ***Stamens*** c. 45, wedge-shaped, c. 1 mm long, anther connective apex flat, often sunken in the middle, inner whorl of stamens with elongated connective apex. ***Carpels*** 5–11, ovary completely covered by long appressed hairs, stigma subglobose, sparsely puberulent. ***Fruits*** with 6–8 shortly stipitate monocarps borne on a globose receptacle, torus 3–4 mm in diameter, sparsely short hairy, stipe 1–4 mm long, 1–2 mm wide, sparsely to densely appressed hairy; fruiting pedicel 4–7 mm long, 1.2–1.5 mm wide, sparsely short hairy; monocarps (excluding stipe) short-cylindrical, 10–22 mm long, 6–11 mm wide, sparsely appressed hairy, green when immature (mature color unknown), apex beaked 1–4 mm long, rarely rounded, base rounded, sometimes with weak constrictions between seeds, pericarp < 0.5 mm thick. ***Seeds*** 1–5 per monocarp, in a single row, discoid, 6–8 mm long, 5–7 mm wide, light brown, surface with pits.

**Figure 3.**
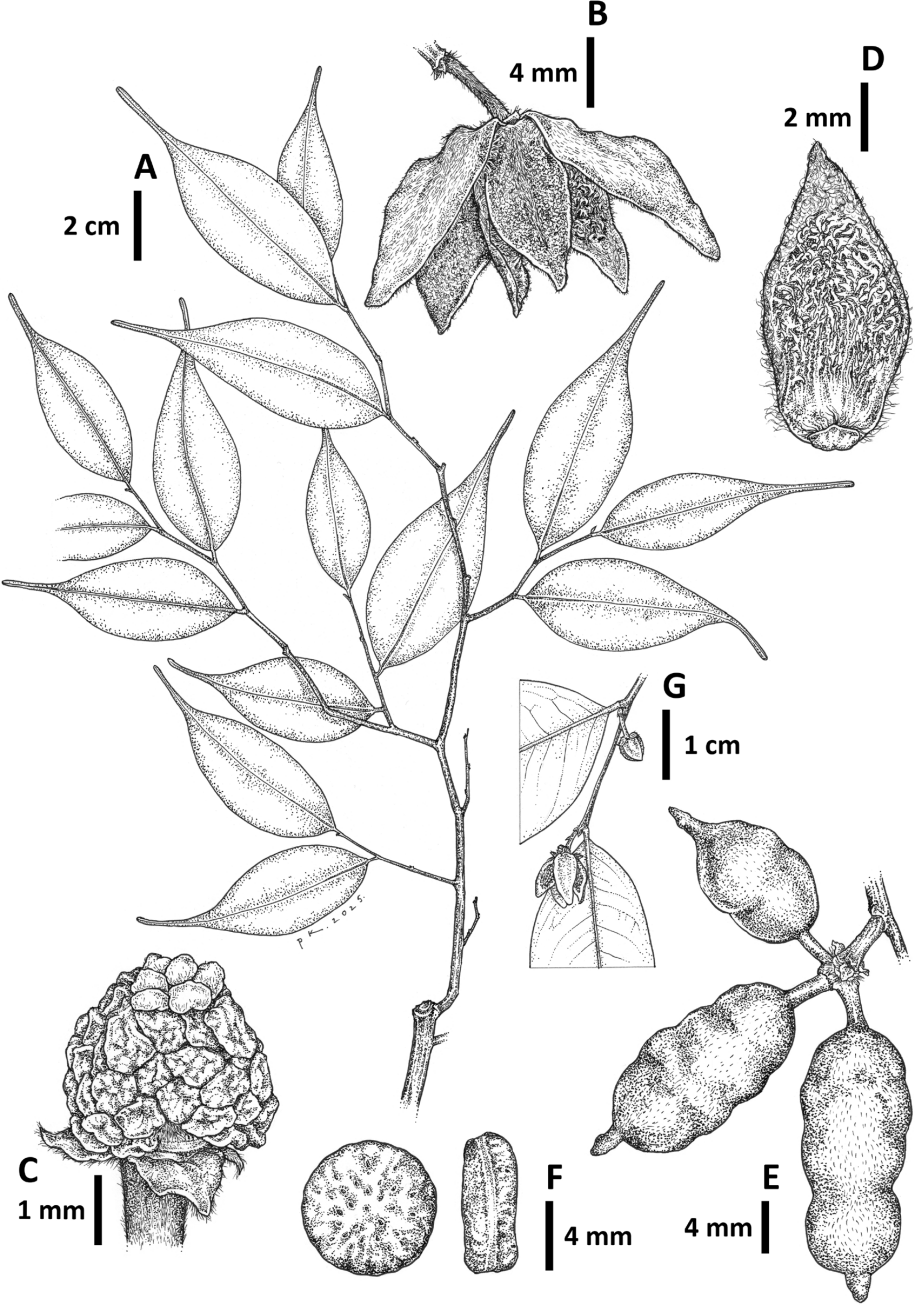
*Meiogyne saundersii* Junhao Chen & M.F.Liu. **A**. Leafy twig; **B**. Flower; **C**. Close-up of stamens and stigmas; **D**. Inner petal, adaxial surface; **E**. Fruit; **F**. Seeds; **G**. Close-up of flowering twig. **A, F**. from *Diwol Sundaling SAN 133028*; **B, D**. from *Hansen 1511*; **C**. from *Goverse & Adriansyah Berau 463*; **E**. from *Leopold Madani SAN 76322*; **G**. from *Argent & Wilkie 966*. Drawn by Cheng Puay Koon.

#### Distribution.

**Figure 4.**
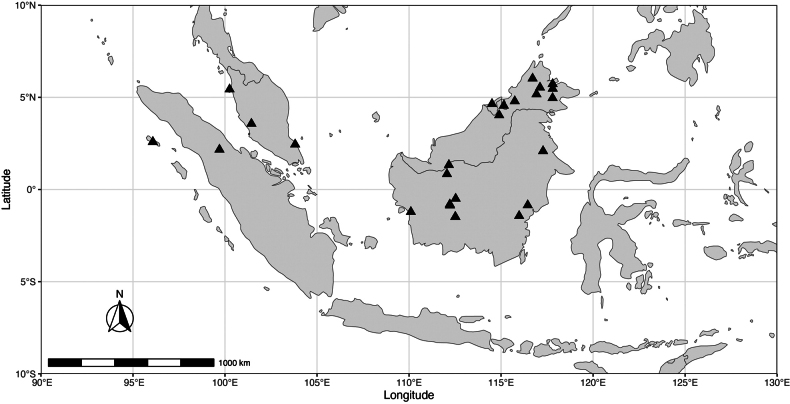
Distribution map of *Meiogyne saundersii* Junhao Chen & M.F.Liu.

Peninsular Malaysia, Sumatra, and Borneo (Brunei, Sabah, Sarawak, and Kalimantan) (Fig. 4).

#### Habitat and ecology.

Lowland dipterocarp forest, secondary forest, sometimes by streams, on sandstone-derived clay soils, rarely on ultramafic substrates, at 20–855 m elevation.

#### Phenology.

Flowers collected from March to November. Fruits collected from July to December.

#### Etymology.

The specific epithet honors Professor Richard Mark Kingsley Saunders, former professor at the University of Hong Kong, for his contribution to Annonaceae systematics, phylogeny, and pollination ecology.

#### Notes.

Van Heusden (1994) noted that *Meiogyne cylindrocarpa* specimens from Borneo and Sumatra differ from the New Guinea specimens in tree size, leaf shape, and flower size but dismissed these as infraspecific variation engendered by habitat, attributing the long caudate leaf apices of the Bornean and Sumatran specimens to a habitat with higher rainfall and concluding that “the differences are not consistent enough to distinguish two species.” However, several molecular phylogenetic studies have suggested that the West Malesian specimens should be regarded as a distinct species (Thomas et al. 2012; Xue et al. 2014, 2021; Jaikhamseub et al. 2022; Liu et al. 2025d). The most densely sampled, well-resolved, and well-supported phylogeny of Liu et al. (2025d) retrieved *Meiogyne cylindrocarpa* (as ‘*Meiogyne cylindrocarpa* (Australia)’) within the Australasia-Pacific clade, whereas *Meiogyne saundersii* (as ‘*Meiogyne cylindrocarpa* (Borneo)’) is nested within the early-divergent basal Asian grade (Fig. 1). Apart from the sharply defined differences mentioned in the diagnosis, *Meiogyne saundersii* also differs from *M. cylindrocarpa* in its monocarp length (10–17 mm long vs. typically 25–35 mm long, excluding stipe) and monocarp apex (typically contracted into a narrow beak 1–4 mm long, rarely rounded vs. typically rounded, rarely mucronately beaked up to 1.5 mm long). The habit of *Meiogyne saundersii* varies from treelets to trees up to 20 m tall, whereas *Meiogyne cylindrocarpa* varies from shrubs to treelets up to 6 m tall. Furthermore, the habitat and climatic niche occupied by the two species differ considerably, with *M. saundersii* occurring in tropical rainforests without a dry season and *M. cylindrocarpa* occupying forests and woodlands with a distinct dry season. The type of *M. cylindrocarpa* was collected from Merauke, a coastal area in Indonesian New Guinea with a pronounced dry season. Other specimens assessed to be conspecific were collected from East Java, the Lesser Sunda Islands, the Maluku Islands, southern coastal parts of New Guinea, the northern part of Western Australia, the northern part of the Northern Territory of Australia, the coastal areas of the Cape York Peninsula, and the Mariana Islands, all of which have a pronounced dry season, with the driest quarter receiving <300 mm rainfall. The geographic distribution of the two species seems sound, as many plant species are restricted to either wet tropics or seasonal tropics (Crisp et al. 2009). Philippine specimens were not included in the concept of *Meiogyne cylindrocarpa* in van Heusden (1994), but Turner (2016) reduced the Philippine *Alphonsea sessiliflora* Merr. to synonymy of *M. cylindrocarpa*. More work is needed to assess whether the Philippine name requires recombination in *Meiogyne*, owing to the scarcity of specimens available. *Oncodostigma wilsonii* Guillaumin from Vanuatu is regarded by van Heusden (1994) as a synonym of *Meiogyne cylindrocarpa*, but we consider the species distinct and transferred it to *Meiogyne* (see below). Apart from being confused with *Meiogyne cylindrocarpa* (e.g., Turner 2014), herbarium specimens of *Meiogyne saundersii* are frequently misidentified as *Alphonsea*, *Polyalthia*, and *Xylopia* species. The morphological differences between *Meiogyne cylindrocarpa*, *Meiogyne saundersii*, and two other species treated below are summarized in Table 1.

**Table 1.** Morphological comparison of *M. cylindrocarpa*, *M. saundersii*, *M. stenophylla*, and *M. wilsonii*.

	*Meiogyne cylindrocarpa*	*Meiogyne saundersii*	*Meiogyne stenophylla*	*Meiogyne wilsonii*
Leaf shape	**Broadly ovate, ovate or elliptic**	Elliptic	Lanceolate	Lanceolate
Leave width	(1.5–)2–4.3 cm	(1.5–)2–3.2(–3.6) cm	**1–1.9 cm**	1.9–3.6 cm
Leaf apex	Acute, rarely shortly acuminate	**Abruptly caudate**	**Attenuate**	Acute
Leaf base	Rounded, rarely cordate, symmetrical to slightly oblique	Cuneate, symmetrical	Cuneate, slightly oblique	**Cordate, oblique**
Outer petal shape	**Broadly ovate**	Triangular	Lanceolate	Triangular to lanceolate
Inner petal shape	**Broadly ovate**	Elliptic to ovate	Lanceolate	Triangular to lanceolate
Inner petal corrugation	**Weakly longitudinally grooved**	**Tentacular**	Weakly warty	Weakly warty
Monocarp shape	Cylindrical	Cylindrical	Cylindrical	**Subglobose**
Monocarp indument	Glabrescent	Sparsely appressed hairy	Sparsely to moderately appressed hairy	Glabrous
Monocarp stipe thickness	1.5–2 mm	1–2 mm	2–3 mm	**4–5 mm**
Pericarp thickness	Up to 1 mm	< 0.5 mm	c. 1 mm	**c. 2 mm**
Geographic distribution	East Java, Lesser Sunda Islands, Maluku Islands, New Guinea, Australia, and Mariana Islands	Peninsular Malaysia, Sumatra, and Borneo	New Guinea	Vanuatu
Habitat	Seasonally dry forests and woodlands at 10–200 m	Rainforests at 20–855 m	Montane forests at 1200 m	Hill forests at 500 m

#### Preliminary conservation status.

The species is rather widespread, with its EOO and AOO estimated at 1,422,770 km^2^ and 100 km^2^, respectively. Moreover, it grows in several protected areas (IUCN Category II: Gunung Mulu National Park, Gunung Palung National Park; IUCN Category Ia: Andulau Forest Reserve, Tangkulap Forest Reserve, Sg Imbak Forest Reserve; IUCN Category VI: Bukit Belata Forest Reserve). Therefore, it is here assessed as Least Concern (LC).

#### Additional specimens examined.

Brunei • Belait: Liang, Andulau Forest Reserve (Sg Liang), Compt. 5, Sg Lumut, 4°38'31"N, 114°30'31"E, 38 m, 16 Aug 2011, *Mhd Ariffin BRUN 23652* (BRUN). Indonesia • Pulow Bulit Tekemeng [Bukit Tekenang?] forest, 10 Oct 1949, *Main (Expedition Polak) 2064* (L). West Kalimantan: Serawai, 3 km southwest of Nanga Jelundung, 0°29'43.5"S, 112°32'3.1"E, 120 m, 31 Oct 1995, *Church et al. 2863* (K) • Ketapang, Gunung Palung National Park, Cabang Panti Research Site, Trail SC, 1°13'S, 110°6'E, 25 m, 9 Mar 1997, *Laman TL 683* (K). East Kalimantan: • Agathis’ Hill, 2^nd^ summit, northwest of Camp Mului, 855 m, s.d., *Raes et al. 706b* (L) • PT-ITCI, near camp Birawa jalan 5200, 491 m, 15 Jan 2002, *Slik IT83-3002* (L) • Berau, INHUTANI I area, near plot 4, 117°17.737'E, 2°05.471'N, 20 m, 3 Mar 1997, *Goverse & Adriansyah Berau 463* (K, L). Central Kalimantan: • Kab. Kotawaringin Timur, km 5 from camp 48, 1°29'S, 112°31'E, c. 50 m, 21 Sep 1996, *Argent & Wilkie 966* (K, L, SING) • Sintang HPH Km 68–70, 0°51'53.6"S, 112°13'29.9"E, 120 m, 16 Apr 1994, *Church et al. 960* (K, SING). Sumatra: • East Coast, Asahan, Masihi Forest Reserve, Oct–Nov 1932, *Krukoff 4170* (L, SING) • Archipel. Ind. Eil Simaloer bij Sumatra, Landschap Tapah, 16 Feb 1920, *Achmad 1693* (L) • ibid., 16 Apr 1920, *Achmad 1801* (L). Malaysia • Sarawak: 4^th^ Division, Gunong Mulu National Park, 530 m, 27 Sep 1976, *Lee S 38066* (K, SAR, U). Sabah: • about 4^th^ mile of Ranau-Poring Road, 22 Jun 1957, *Sinclair et al. 9277* (L, SING) • Lahad Datu, Danum Valley, s.d., *Ridsdale DV-M11930* (L) • Lahad Datu, Danum Valley and environs, s.d., *Anonymous 1/1930* (SAN) • ibid., *Anonymous R3197* (SAN) • Lamag, Karamuak, Sg Korong, 500 ft, 6 Sep 1973, *Leopold Madani SAN 76322* (K, L, SAN) • Sandakan, Segaliud Lokan, 11 Jul 1988, *Julius et al. SAN 124229* (SAN) • Sandakan, Tangkulap Forest Reserve, 7 Dec 1985, *Sigin et al. SAN 108019* (SAN) • Sipitang, 6 miles from Mandulang road to Maligan, 7 Sep 1983, *Lee & Dewol SAN 69854* (K, L, SAN) • Telupid, Bukit Tawai Forest Reserve, near sawmill, 5°31'N, 117°04'E, 100 m, 10 Apr 1994, *Mat Salleh & Zainuddin KMS 3440* (KEP, L, SAN) • Telupid, Bukit Tawai Forest Reserve, behind Golden Apex Sawmill, 10 Apr 1994, *Zainuddin et al. 5007* (K, KEP, SAN) • Tongod, South of Sg Imbak Forest Reserve, 2 Jul 2000, *Diwol Sundaling SAN 133028* (K, SAN, SAR, SING). Penang: • West Hill, 1888, *Curtis s.n*. (SING). Selangor: • Hulu Selangor, Bukit Belata Forest Reserve, survey trail parallel to main trunk road B33, 3°34.06'N, 101°26.26'E, 59 m, 25 May 2016, *Chew et al. FRI 73143* (KEP). Johor: • Mersing, 20 Sep 1994, *Wiart & Teo KL 4405* (KEP).

### Meiogyne stenophylla

Taxon classificationPlantaeMagnolialesAnnonaceae

M.F.Liu & Munzinger sp. nov.

89B65B57-CCEF-5620-9EBC-2EFCAEA6AA93

urn:lsid:ipni.org:names:77377869-1

Figs 2C, 2D, 5

‘Meiogyne sp. PNG’ in Liu et al. (2025d); ‘Meiogyne sp. 1’ in Munzinger et al. (2020).

#### Type.

Papua New Guinea • Madang Province: Bismarck Range, Mt. Wilhelm, Base Camp, 1200 m, 5°43'51.59"S, 145°16'24.93"E, 6 Nov 2012, fl., fr., *J. Munzinger, J.-F. Molino, K. Molem & J.-C. Pintaud 6945* (holotype: P [P02275660!]; isotypes: LAE!, P [P02433813!]).

**Figure 5.**
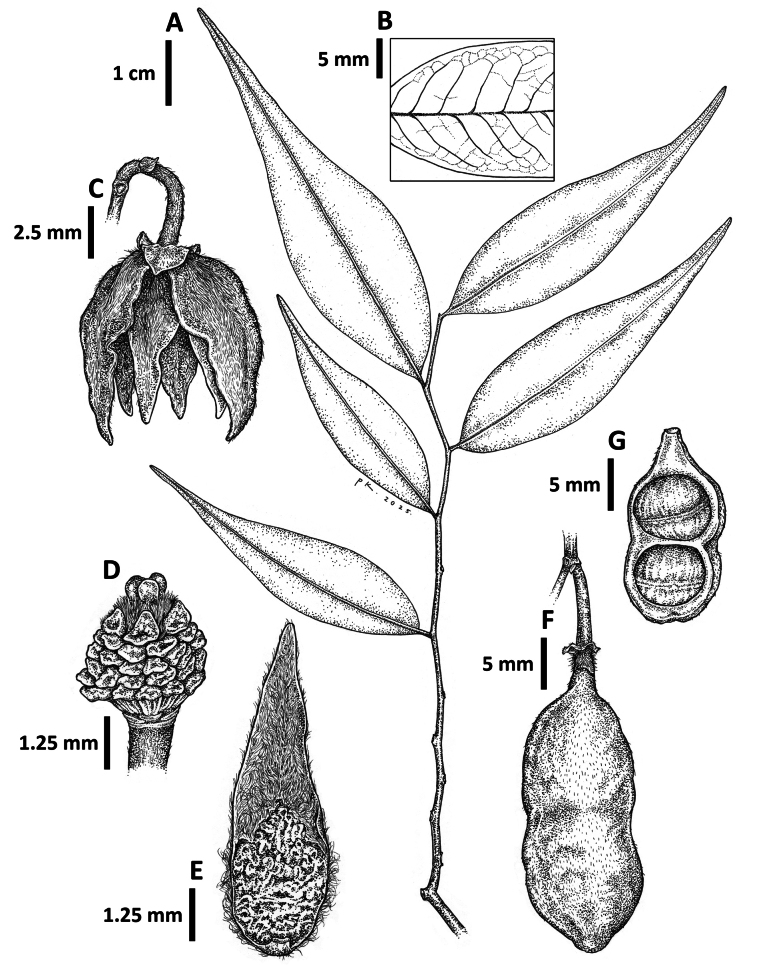
*Meiogyne stenophylla* M.F.Liu & Munzinger. **A**. Leaf twig; **B**. Close-up of leaf; **C**. Flower; **D**. Close-up of stigmas and stamens; **E**. Inner petal, adaxial surface; **F**. Fruit; **G**. Fruit cut open, showing seeds. All from the type, *Munzinger et al. 6945*. Drawn by Cheng Puay Koon.

#### Diagnosis.

Unique in the genus in its extremely narrow leaves (< 2 cm wide) with an attenuate apex. Similar to *Meiogyne cylindrocarpa* in its relatively short leaves (typically < 10 cm long), indistinct leaf venation, and cylindrical monocarps, but differs in its lanceolate (vs. broadly ovate) petals and weakly warty (vs. weakly longitudinally grooved) outgrowth on the adaxial surface of inner petals.

#### Description.

Shrubs. ***Twigs*** densely appressed hairy when young, becoming glabrous with inconspicuous lenticels when old. ***Leaves*** chartaceous, blades lanceolate, 3.8–7.4 cm long, 1–1.9 cm wide, base cuneate and slightly oblique, apex attenuate, glabrous adaxially, sparsely appressed hairy abaxially; midrib slightly impressed adaxially, raised abaxially, secondary veins and tertiary venation indistinct; petiole 0.5–2.5 mm long. ***Inflorescences*** axillary, 1-flowered; pedicel 5–7 mm long, 1 mm thick, densely suberect hairy, with 1–3 small bract(s) at the proximal end of the pedicel. ***Buds*** conical, apex acute. ***Sepals*** free or connate at base, broadly ovate, c. 1 mm long, c. 2 mm wide, apex acute, densely appressed hairy abaxially. ***Petals*** pale green to yellowish green, lanceolate, apex narrowly acute; outer petals 9–10 mm long, 2–3 mm wide, densely appressed hairy on both sides; inner petals 7–8 mm long, 2–2.5 mm wide, densely appressed hairy on both sides except the corrugated base, with a dense tuft of curly hairs on the margin at the base, adaxial corrugation weakly warty, extending 1/3 from the base, glabrous. ***Stamens*** c. 40, wedge-shaped, c. 1 mm long, anther connective apex slightly sunken in the middle, inner whorl of stamens elongated, sometimes oblique. ***Carpels*** c. 3, ovary completely covered by long appressed hairs, stigma globose, sparsely puberulous. ***Fruits*** with c. 3 monocarps borne on a globose receptacle, torus c. 2 mm in diameter, erect hairy, stipe 2–4 mm long, 2–3 mm wide, sparsely to moderately appressed hairy; fruiting pedicel c. 9 mm long, c. 1 mm wide, sparsely to moderately suberect hairy; monocarps (excluding stipe) cylindrical, 19–28 mm long, 9–11 mm wide, sparsely to moderately appressed hairy, green when immature (mature color unknown), apex beaked 1–4 mm long, rarely rounded, base rounded, with weak constrictions between seeds, pericarp c. 1 mm thick. ***Seeds*** 1–3 per monocarp, in a single row, globose, ellipsoid, or discoid, 7–9 mm long, 3–6 mm wide, light brown, surface without pits.

#### Distribution.

**Figure 6.**
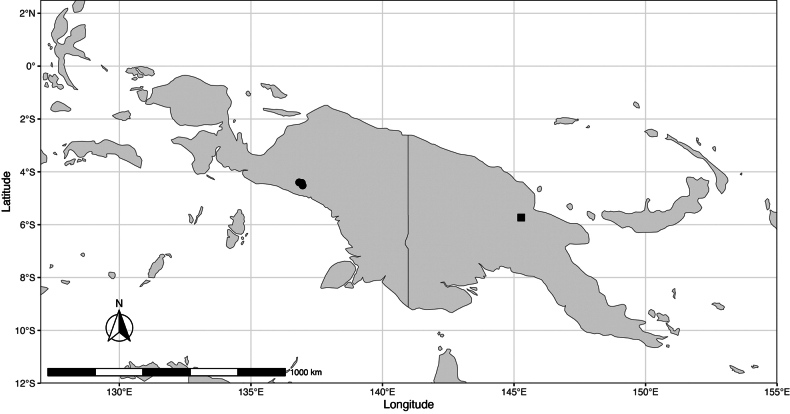
Distribution map of *Meiogyne stenophylla* M.F.Liu & Munzinger (square) and *Monoon papuanum* (I.M.Turner & Utteridge) M.F.Liu & Junhao Chen (circle).

Papua New Guinea (Fig. 6).

#### Habitat and ecology.

Montane forest at an elevation of 1200 m.

#### Phenology.

Flowers and fruits collected in November.

#### Etymology.

The name alludes to the narrow leaves of this species.

#### Notes.

This species is known only from the lower montane forests on the northern side of the Central Cordillera in Papua New Guinea (Madang Province). A molecular phylogenetic study (Liu et al. 2025d) retrieved *Meiogyne stenophylla* (as ‘*Meiogyne* sp. PNG’) as deeply nested within the Australasia-Pacific clade (APC; c. 19 species), whereas *Meiogyne cylindrocarpa* is the earliest diverging lineage of the APC (Fig. 1). Specifically, *Meiogyne stenophylla* is sister to a clade that comprises all *Meiogyne* species in Vanuatu, Fiji and Tonga (Liu et al. 2025d; Fig. 1). See Table 1 for a morphological comparison between *Meiogyne stenophylla* and morphologically similar congeners.

#### Preliminary conservation status.

This species is known from only a single gathering. The New Guinea highlands are very poorly explored and collected (Munzinger et al. 2020), so it is unclear whether the species is more widely distributed than the current data suggest. Hence, it is assessed here as Data Deficient (DD).

### Meiogyne wilsonii

Taxon classificationPlantaeMagnolialesAnnonaceae

(Guillaumin) M.F.Liu & Munzinger comb. nov.

72BA8028-FC34-5E40-BD3F-39330674DDC4

urn:lsid:ipni.org:names:77377870-1

Fig. 2E, F

Oncodostigma wilsonii Guillaumin, J. Arnold Arbor. 12: 224 (1931). Type: VANUATU (ex New Hebrides) • Aneityum Island, Anelgauhat Bay, lower hills in forest, 500 m, Sep 1929, *J.P. Wilson 986* (lectotype P [P00636932!], designated here; isolectotypes A [A00066603!, A00066604!], B [B_10_0272878!], BRI [BRI-AQ0332769!], K [K000691928!], LA [LA00000046!], NY [NY00026145!]). [Basionym]
Meiogyne cylindrocarpa auct. non (Burck) Heusden: van Heusden, Blumea 38: 499 (1994), *pro parte*.
‘Meiogyne cylindrocarpa (Vanuatu)’ in Liu et al. (2025d).

#### Notes.

Van Heusden (1994) considered this species a synonym of *Meiogyne cylindrocarpa* but noted that the Vanuatuan specimens differed in having globose (vs. cylindrical) monocarps. However, the immature monocarps of *Meiogyne wilsonii* are cylindrical (Fig. 2F; *Tuiwawa & Munzinger 2956*, *Munzinger 3589*), which might have influenced van Heusden’s decision to merge it with *M. cylindrocarpa*. Our observations additionally revealed that *Meiogyne wilsonii* has lanceolate leaf blades and a thick pericarp (c. 2 mm), whereas *Meiogyne cylindrocarpa* has broadly ovate, ovate, or elliptic leaf blades and a thin pericarp (up to 1 mm). A molecular phylogenetic study (Liu et al. 2025d) indicated that *Meiogyne wilsonii* (as ‘*M. cylindrocarpa* (Vanuatu)’) is more closely related to the Fijian and Tongan *Meiogyne* species than to *M. cylindrocarpa* sampled from Australia, which is morphologically similar to the type of *M. cylindrocarpa* from New Guinea (Fig. 1). *Meiogyne wilsonii* is most similar to *Meiogyne habrotricha* (A.C.Sm.) B.Xue & R.M.K.Saunders from Fiji in its subglobose monocarps, cordate leaf base, and pubescent branchlets but differs in its glabrous (vs. copiously velutinous-puberulent) monocarp and longer and thicker monocarp stipes (2.5–3.5 × 4–5 mm vs. 1–2 × 2–3 mm). See Table 1 for a morphological comparison between *Meiogyne wilsonii* and other morphologically similar species.

Van Heusden (1994) and Turner (2018) erroneously indicated that the P sheet is the holotype of *Oncodostigma wilsonii*, possibly because van Heusden annotated the P sheet as a holotype. However, Guillaumin (1931) did not indicate a particular herbarium in which the type was deposited in the protologue, and he manually annotated “*Oncodostigma wilsonii* Guillaumin, sp. nov.” on the A, BRI, and P sheets, suggesting that he likely studied all of them. The P sheet is selected here as the lectotype, as it is the only specimen with both mature flowers and fruits on the same sheet.

#### Preliminary conservation status.

**Figure 7.**
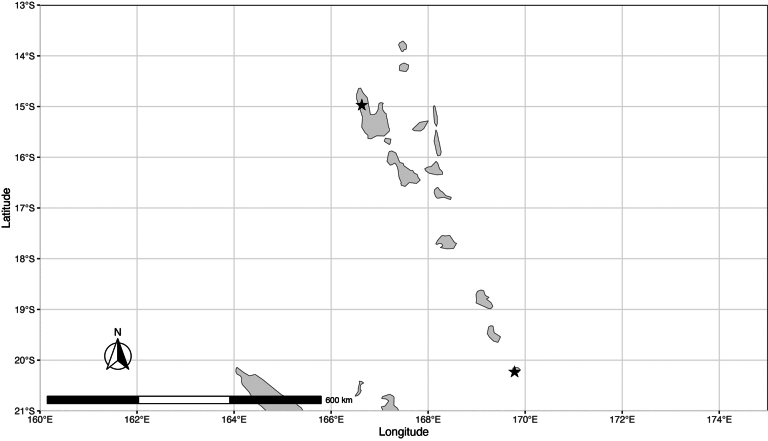
Distribution map of *Meiogyne wilsonii* (Guillaumin) M.F.Liu & Munzinger (star).

This species is known only from the Vanuatu archipelago, where it is recorded from the northern island Espiritu Santo and the southernmost island Aneityum but not from the intervening islands (Fig. 7). Like New Guinea, Vanuatu is a poorly collected region (Plunkett et al. 2022), suggesting that the current data are likely insufficient for assessing its true distribution. Therefore, it is assessed here as Data Deficient (DD).

### Monoon papuanum

Taxon classificationPlantaeMagnolialesAnnonaceae

(I.M.Turner & Utteridge) M.F.Liu & Junhao Chen comb. nov.

7C38266E-9C1E-52C4-99FE-8C7338B3BF90

urn:lsid:ipni.org:names:77377871-1

Meiogyne papuana I.M.Turner & Utteridge, Kew Bulletin 70(2)-27: 2 (2015). Type: Indonesia • Papua Province: Mimika Regency, PT-Freeport Indonesia Concession of Work area, Kuala Kencana, grounds of Rimba Irian Golf Club, 4°24'S, 136°52'E, 7 Apr 2000, *T. Triono, T.M.A. Utteridge, R.J. Johns & O. Kasmin 121* (holotype K [K000260884!, K000260885! – a single specimen over 2 sheets]; isotypes A [A00549712!], BISH, BO, BR, CANB, Freeport, KYO, L [L.3728206!], LAE, MAN, MO, NSW, SAN, SING [SING0404965!], TI). [Basionym]

#### Notes.

Examination of the protologue and type specimens of this species revealed that it possesses decurrent secondary veins, a single seed per monocarp, and elongated stigmas; these characters suggest that it should be placed in the genus *Monoon* (Xue et al. 2012). Liu et al. (2025d) sampled the paratype *Dransfield 7692* (K!), and the phylogeny revealed that *Meiogyne papuanum* is nested within *Monoon* with maximum support. The majority of *Monoon* species in New Guinea have narrowly triangular to linear petals that are smooth on both sides (Ezedin 2025), but *Monoon papuanum* has ovate-triangular petals with verrucose bases on the adaxial surface of the inner petals. It is similar to *Monoon pachypetalum* I.M.Turner & Utteridge and *Monoon salomonicum* I.M.Turner & Utteridge in having broadly ovate to ovate-lanceolate petals and warty bases on the adaxial surface of the inner petals. However, *Monoon papuanum* is distinct from these species in its glabrous (vs. adpressed hairy or tomentose) monocarps. Additionally, *Monoon papuanum* has longer fruiting pedicels (up to 40 mm) than *Monoon pachypetalum* (up to 25 mm) and *Monoon salomonicum* (up to 18 mm).

#### Preliminary conservation status.

This species was provisionally assessed as Endangered (EN B1ab(iii)) by Turner and Utteridge (2015).

## Supplementary Material

XML Treatment for Meiogyne saundersii

XML Treatment for Meiogyne stenophylla

XML Treatment for Meiogyne wilsonii

XML Treatment for Monoon papuanum

## Additional information

### Use of AI

No use of AI was reported.

### Author ORCIDs

Ming-Fai Liu https://orcid.org/0000-0002-4732-2512

Jérôme Munzinger https://orcid.org/0000-0001-5300-2702

Junhao Chen https://orcid.org/0000-0001-8822-169X

### Data availability

All of the data that support the findings of this study are available in the main text.
